# Effect of Pediatric Rehabilitation on Children With Viral Encephalitis: A Case Report

**DOI:** 10.7759/cureus.57239

**Published:** 2024-03-30

**Authors:** Anushka M Biyani, Vasanth Sharath, Tanvi S Varma

**Affiliations:** 1 Department of Pediatric Physiotherapy, Ravi Nair Physiotherapy College, Datta Meghe Institute of Higher Education and Research, Wardha, IND; 2 Department of Cardiovascular and Respiratory Physiotherapy, Ravi Nair Physiotherapy College, Datta Meghe Institute of Higher Education and Research, Wardha, IND

**Keywords:** cognitive impairment, viral infection, central nervous system, inflammation, encephalitis

## Abstract

Viral encephalitis poses a significant threat to public health, particularly affecting children and the elderly. We present a case of a 1.5-year-old child with viral encephalitis, characterized by sudden convulsions following a bout of cold and fever. Comprehensive physiotherapy rehabilitation was initiated, focusing on improving mobility, strength, and functional abilities. The interventions included caregiver education, range of motion exercises, strengthening exercises, mobility training, and task-oriented activities. After treatment, there was a notable improvement in the child's clinical outcomes, as evidenced by a reduction in weakness, enhanced functional mobility, and improved scores on outcome measures such as the Pediatric Cerebral Performance Category and Functional Mobility Scale. This case highlights the importance of early physiotherapy intervention in pediatric viral encephalitis to mitigate long-term complications and optimize functional outcomes.

## Introduction

Viral encephalitis, which results from inflammation of the brain parenchyma due to viral infection, represents the most prevalent form of encephalitis. Viruses typically enter the body outside the central nervous system and travel via the bloodstream to the brain and spinal cord. Younger individuals are more susceptible to viral encephalitis compared to the elderly. While viral causes account for about 70% of diagnosed cases, the etiology of many instances remains unknown despite extensive investigation. This condition affects 3.5-7.5 individuals per 100,000, with the highest incidence observed among the young and elderly, remaining a significant cause of acute neurological impairment and lasting disability, especially in children [1-5].

Numerous viral agents, including arboviruses, herpesviruses, and coronaviruses, have been implicated in causing encephalitis. Once a neurotropic virus infiltrates the brain parenchyma, resident cells, such as neurons and glial cells, become infected, triggering an inflammatory response that can lead to neuronal damage [6-8]. Even after the resolution of the viral infection, persistent immune activation may contribute to long-term neurological and cognitive impairments [9,10]. When encephalitis is suspected, it is recommended to conduct diagnostic tests, such as brain MRI, EEG, and lumbar puncture.

Lumbar puncture helps analyze cerebrospinal fluid for signs of infection, inflammation, and other abnormalities. MRI provides detailed images of the brain to identify structural changes or abnormalities caused by the viral infection. EEG measures electrical activity in the brain, helping to detect abnormal patterns or seizures. In viral encephalitis (VE), CSF typically shows elevated protein levels, nearly normal glucose levels, and mild-to-moderate mononuclear pleocytosis, primarily lymphocytic but occasionally neutrophilic, especially in the early stages [11-14]. Normal CSF white blood cell (WBC) counts in infants are higher than in adults, with a 95th percentile cutoff of no more than 19 WBCs/μL for infants under one month of age and no more than 9 WBCs/μL for infants aged one to two months. Repeat lumbar puncture should be considered if symptoms persist or worsen [15].

Effective rehabilitation for encephalitis patients requires a comprehensive approach that addresses the complex medical, behavioral, social, emotional, and cognitive challenges faced by patients and their families. For pediatric patients with viral encephalitis, physical therapy rehabilitation is particularly important as it addresses the functional and physical difficulties resulting from brain inflammation. The goal of physiotherapy is to improve mobility, motor coordination, strength, flexibility, and balance [16,17]. Physiotherapy interventions have the potential to enhance functional abilities, muscle strength, and range of motion in pediatric cases of viral encephalitis. Early initiation of physiotherapy is essential in pediatric patients to reduce the risk of long-term complications, improve quality of life, and facilitate overall recovery. Rehabilitation for viral encephalitis patients requires a comprehensive approach addressing medical, behavioral, social, emotional, and cognitive aspects. Physical therapy plays a crucial role in pediatric cases, aiming to improve mobility, motor coordination, strength, flexibility, and balance [18].

## Case presentation

Patient information

A 1.5-year-old patient was apparently alright one month ago when he developed a cold, cough, and fever. He was then brought by his parents to Acharya Vinoba Bhave Rural Hospital (AVBRH). The cough was dry in nature and insidious in onset. The fever was moderate and relieved on medications. It was not associated with chills and rigors. No complaints of vomiting or loose stools, and there was no decrease in activity. The patient then underwent a sudden episode of convulsions, for which investigations like contrast-enhanced magnetic resonance imaging (CEMRI) and magnetic resonance angiography (MRA) brain were done. The investigations revealed viral encephalitis and the patient was admitted to the ICU at Acharya Vinoba Bhave Rural Hospital for the treatment. Presently, according to the patient’s caregiver, the patient is having bilateral lower limb and upper limb weakness and pain in extremities. Patient's caregiver gives the history of episodes of pneumonia and the patient was admitted for the same at AVBRH in September. He was then referred for further physiotherapy management.

On examination

Before commencing the examination, the informed consent was obtained, following which a thorough examination was conducted. During the examination, he was hemodynamically stable. Physically, the patient presented an ectomorphic physique. On auscultation, the chest was bilaterally symmetrical along with an abdominothoracic breathing pattern. There was no murmur or crepitus audible during auscultation, and bilateral air entry was also equal. The patient's current anthropometric measurements are 74 cm in height, 7 kg in weight, with a BMI of 12.78 kg/m^2^. The chest circumference is 45 cm and the head circumference is 43 cm.

The child achieved various developmental milestones at different ages. He gained head control at three months, rolled over at four months, and sat independently at 11 months. Additionally, he began creeping at 11 months, crawling at 12 months, and standing with support at 12 months. The child took their first steps with support at 12 months and started walking without support at 13 months. As they grew, their motor skills improved, reaching milestones such as balancing at 17 months, climbing at 20 months, and walking confidently at 21 months. By 24 months, they demonstrated advanced balancing skills. These achievements in gross and fine motor skills, along with language and social development, reflect a typical progression in a child's early years.

Clinical investigations

**Figure 1:**
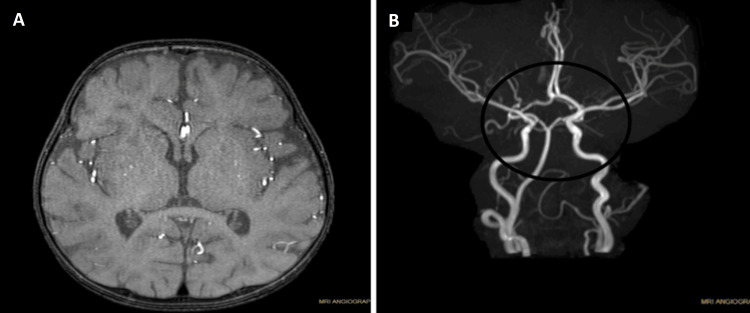
CEMRI and MRA of the patient. (A) CEMRI findings indicating multiple non-enhancing areas in various regions of the brain suggesting the presence of viral encephalitis and (B) the finding of hypoplasia on MRA brain suggesting a congenital variation in the development of the arterial system. CEMRI: contrast-enhanced magnetic resonance imaging; MRA: magnetic resonance angiography

Investigations like contrast-enhanced magnetic resonance imaging (CEMRI) reveal evidence of multiple non-enhancing areas noted in bilateral cerebral and cerebellar hemispheres predominantly in white matter, also in gangliocapsular region, left anterior thalamus, and bilateral posterior central gyrus. Left anterior thalamus and magnetic resonance angiography (MRA) (Figures 1A, 1B).

Physiotherapy intervention

A multifaceted physiotherapy approach was utilized to address motor impairments, balance deficits, and functional limitations (Table 1). Various outcome measures including motor function assessments, balance scales, and quality of life metrics were employed to monitor the patient's progress throughout the rehabilitation process (Table 2). The findings underscore the significance of tailored physiotherapy interventions and comprehensive assessment tools in optimizing functional outcomes for pediatric patients facing the complexities of viral encephalitis [19].

**Table 1:** Physiotherapy rehabilitation protocol.

S. no.	Goals	Intervention	Exercise dose
1	Patient caregiver education	To educate caregiver about the condition and explain significance of physical therapy rehabilitation	Counselling session (1-2 times)
2	Weakness in upper limb and lower limb B/L	PNF (rhythmic initiation) D1 flexion, extension	(20 reps × 2 sets) 2 times
3	Reduced strength	Climbing and swinging on playground equipment	3-4 times in a day
4	To improve mobility	Rolling facilitation	10 reps × 2 times
		Supine to sitting	
		Sit to stand	
5	To improve functional activities	Task-oriented activities	3-4 times in a day
		Play full activities	

**Table 2:** Pre- and post-treatment outcomes. Functional mobility scale range: 1=uses wheelchair, 2=uses a walker or frame, 3=uses crutches, 4=uses sticks (one or two), 5=independent on level surfaces, 6=independent on all surfaces; Face, Legs, Activity, Cry, Consolability (FLACC) pain scale: 0=relaxed and comfortable, 1-3=mild discomfort, 4-6=moderate pain; and Pediatric Cerebral Performance Category Scale: score 0=no neurological deficit, score 1: slight neurological or functional impairment, score 2: moderate neurological or functional impairment, score 3: severe neurological or functional impairment, score 4: unresponsive, with no meaningful interaction with the environment, score 5: complete cessation of cerebral and brainstem function

Outcome measures	Pre-treatment score	Post-treatment score (1 month follow-up)
Pediatric cerebral performance category	Score -3	Score -2
Functional mobility scale	Rating 1	Rating 3
FLACC pain scale	Score 6/10	Score 3/10

## Discussion

Pediatric rehabilitation for viral encephalitis with hypoplastic left vertebral artery is a complex and challenging endeavor that requires a multidisciplinary approach. Viral encephalitis, particularly in children, can lead to significant neurological deficits and impairments in motor function, cognition, and behavior. The study provides valuable insights into the physiotherapy rehabilitation of a pediatric patient with viral encephalitis. The comprehensive approach to rehabilitation, focusing on caregiver education, addressing weakness and reduced strength, improving mobility and gait, and enhancing functional activities, highlights the importance of early and multidisciplinary intervention in such cases. The utilization of proprioceptive neuromuscular facilitation (PNF) techniques, strengthening exercises, and task-oriented activities demonstrates a tailored approach to address specific impairments associated with viral encephalitis, such as weakness and mobility limitations.

The inclusion of activities like climbing, swinging, and walking with support not only targets physical function but also promotes engagement and participation in meaningful activities for the child. When compounded with anatomical variations such as a hypoplastic left vertebral artery, the rehabilitation process becomes even more intricate. This study underscores the importance of tailored rehabilitation interventions designed to address the specific needs and deficits of each individual patient. In the case of viral encephalitis, early rehabilitation efforts focus on mitigating the acute effects of the infection, such as managing seizures, reducing inflammation, and providing supportive care to prevent further neurological damage [19,20].

Once the acute phase has stabilized, the rehabilitation team can begin implementing a comprehensive treatment plan aimed at maximizing the child's functional independence and quality of life. This may include physical therapy to improve strength, balance, and coordination, occupational therapy to enhance activities of daily living and fine motor skills, speech therapy to address communication difficulties, and neuropsychological interventions to support cognitive and behavioral challenges. In the context of a hypoplastic left vertebral artery, additional considerations may be necessary to ensure the safety and efficacy of rehabilitation interventions. Close monitoring of blood flow and cerebral perfusion is crucial, and modifications to therapy techniques may be required to minimize the risk of vascular compromise or ischemic events.

The outcome measures, including the Pediatric Cerebral Performance Category, Functional Mobility Scale, and FLACC Pain Scale, provide objective criteria to evaluate the effectiveness of the physiotherapy intervention. The improvement observed in these measures post-treatment indicates the positive impact of rehabilitation on the patient's functional outcomes and quality of life. Furthermore, the discussion emphasizes the significance of early diagnosis and management of viral encephalitis, highlighting the role of diagnostic tests such as brain MRI, EEG, and lumbar puncture. It also underscores the need for a multidisciplinary approach to rehabilitation, considering the complex medical, behavioral, social, emotional, and cognitive issues associated with encephalitis. Overall, this study underscores the importance of physiotherapy rehabilitation in optimizing outcomes for pediatric patients with viral encephalitis, emphasizing the need for early intervention and comprehensive care to facilitate recovery and minimize long-term complications.

Engaging caregivers in the rehabilitation process, providing education on the child's condition and prognosis, and offering emotional support can help optimize outcomes and promote long-term success. Overall, this study highlights the intricate interplay between viral encephalitis, vascular anomalies, and rehabilitation interventions in pediatric patients. By employing a multidisciplinary approach that addresses the unique needs of each child, rehabilitation professionals can play a pivotal role in promoting recovery and maximizing functional outcomes.

## Conclusions

This study highlights the importance of physiotherapy rehabilitation in pediatric patients with viral encephalitis. Early initiation of physiotherapy interventions can significantly improve functional abilities, muscle strength, and range of motion, thus aiding in the overall recovery and quality of life of the patient. The comprehensive approach of physiotherapy acknowledges the complex medical, behavioral, social, emotional, and cognitive issues associated with encephalitis, ensuring a holistic rehabilitation process. It is crucial for healthcare providers to recognize the significance of physiotherapy in managing the sequelae of viral encephalitis and to integrate it into the multidisciplinary care of affected patients.

## References

[1] Said S, Kang M (2023). Viral encephalitis. StatPearls [Internet].

[2] Bale JF Jr (2015). Virus and immune-mediated encephalitides: epidemiology, diagnosis, treatment, and prevention. Pediatr Neurol.

[3] De Chiara G, Marcocci ME, Sgarbanti R (2012). Infectious agents and neurodegeneration. Mol Neurobiol.

[4] Aneja S, Sharma S (2019). Diagnosis and management of acute encephalitis in children. Indian J Pediatr.

[5] Clé M, Eldin P, Briant L (2020). Neurocognitive impacts of arbovirus infections. J Neuroinflammation.

[6] Bohmwald K, Andrade CA, Gálvez NM, Mora VP, Muñoz JT, Kalergis AM (2021). The causes and long-term consequences of viral encephalitis. Front Cell Neurosci.

[7] Sonneville R, Klein I, de Broucker T, Wolff M (2009). Post-infectious encephalitis in adults: diagnosis and management. J Infect.

[8] Kramer AH (2013). Viral encephalitis in the ICU. Crit Care Clin.

[9] Bassetti C, Sturzenegger M (1999). Viral encephalitis. [Article in German]. Ther Umsch.

[10] Sudhir CS, Sharath HV (2023). A brief overview of recent pediatric physical therapy practices and their importance. Cureus.

[11] Granerod J, Ambrose HE, Davies NW (2010). Causes of encephalitis and differences in their clinical presentations in England: a multicentre, population-based prospective study. Lancet Infect Dis.

[12] Hjalmarsson A, Blomqvist P, Sköldenberg B (2007). Herpes simplex encephalitis in Sweden, 1990-2001: incidence, morbidity, and mortality. Clin Infect Dis.

[13] Sheikh SS, Sharath HV, Seth NH (2024). A rare case report on postoperative rehabilitation in Hirschsprung disease. Cureus.

[14] George BP, Schneider EB, Venkatesan A (2014). Encephalitis hospitalization rates and inpatient mortality in the United States, 2000-2010. PLoS One.

[15] Costa BK, Sato DK (2020). Viral encephalitis: a practical review on diagnostic approach and treatment. J Pediatr (Rio J).

[16] Nerkar S 4th, Sharath HV, Kochar SS, Bhoyar SS (2023). Impact of neurological rehabilitation in autoimmune encephalopathy: a case report. Cureus.

[17] Tunkel AR, Glaser CA, Bloch KC (2008). The management of encephalitis: clinical practice guidelines by the Infectious Diseases Society of America. Clin Infect Dis.

[18] Thompson C, Kneen R, Riordan A, Kelly D, Pollard AJ (2012). Encephalitis in children. Arch Dis Child.

[19] Huttunen P, Lappalainen M, Salo E, Lönnqvist T, Jokela P, Hyypiä T, Peltola H (2009). Differential diagnosis of acute central nervous system infections in children using modern microbiological methods. Acta Paediatr.

[20] Romero JR, Newland JG (2003). Viral meningitis and encephalitis: traditional and emerging viral agents. Semin Pediatr Infect Dis.

